# Morphological and Bactericidal Effects of Different Antibiotics on *Helicobacter pylori*

**DOI:** 10.5812/jjm.8704

**Published:** 2014-01-01

**Authors:** Jamshid Faghri, Farkhondeh Poursina, Sharareh Moghim, Hamid Zarkesh Esfahani, Bahram Nasr Esfahani, Hossein Fazeli, Nasrin Mirzaei, Azam Jamshidian, Hajieh Ghasemian Safaei

**Affiliations:** 1 Department of Microbiology, Faculty of Medicine, Isfahan University of Medical Sciences, Isfahan, IR Iran; 2 Department of Immunology, Faculty of Medicine, Isfahan University of Medical Sciences, Isfahan, IR Iran; 3 Department of Biology, Islamic Azad University, Tonekabon branch, Tonekabon, IR Iran

**Keywords:** *Helicobacter pylori*, Coccoid Bacteria, Antibiotics, Susceptibility

## Abstract

**Background:**

*Helicobacter pylori* (*H. pylori*) is a spiral Gram negative bacteria that can transform to the coccoid form in adverse conditions.

**Objectives:**

The aim of this study was to determine the *in vitro* morphological and bactericidal effects of metronidazole, amoxicillin and clarithromycin on *H. pylori*.

**Materials and Methods:**

The standard strain 26695 of *H. pylori* was cultured on Brucella agar (BA) and the minimum inhibitory concentrations (MICs) of three antibiotics were determined by E-test method. The bacteria were exposed to antibiotics at 1/2 MIC, MIC and 2X MIC concentrations in Brucella broth (BB). Induced coccoid forms were confirmed by Gram staining and light microscopy. The viability of cells as well as the susceptibility of viable coccoids to antibiotics were examined using the flow cytometry method.

**Results:**

All of the three antibiotics at sub-MIC induced coccoid forms. The highest rates of coccoids (> 90%) were induced at 0.008 μg/mL concentration (1/2 MIC) of amoxicillin, 72 hours postexposure. Metronidazole and clarithromycin with 1/2 MIC (0.5 and 0.125 µg/mL respectively) induced lower rates of coccoid forms (60% and 40% respectively). Potent bactericidal effects on coccoids were observed with Metronidazole at 2X MIC and clarithromycin at MIC (0.25 µg/mL) (80 - 90%). Amoxicillin with MIC and 2X MIC had no bactericidal effect on coccoid forms.

**Conclusions:**

Despite the good *in vitro* bactericidal effect of amoxicillin on spiral forms of *H. pylori*, this antibiotic has little effect on induced coccoids that may develop after the inappropriate *in vivo* antibacterial treatment. Hence, for successful therapy, it is essential not only to eradicate the spiral forms, but to eliminate the viable coccoids.

## 1. Background

*Helicobacter pylori* is a Gram negative curved bacterium, recognized as the major cause of gastritis, peptic ulcer, and gastric cancer in humans (1, 2). The majority of bacteria in the gastric mucosal layer are spiral, but in unfavorable conditions, *H. pylori* can convert to coccoid form (3). Morphological changes are responses to physical and chemical stresses such as increased oxygen tension, pH changes, extended *in vitro* incubation, and exposure to antibiotics (4). Kusters et al. have reported that this conversional form is a manifestation of the cell death (5). Coccoid forms have been divided into two types, A and B, by electron microscopy. Type A is irregular with rough surface that is considered dead, and type B is smoother and smaller with complete membrane as live cells (6). Various authors have shown that coccoid forms of *H. pylori* can be viable and are manifestations of cell adaptation to the environment. They named these forms of bacteria as VBNC (viable but nonculturable) (7). After chemical therapy, *H. pylori* organisms may have rarely survived as coccoid forms, and it is suspected that these forms play a role in the transmission and relapse of the infection in human (8, 9). In the gastric tissues, coccoid forms of *H. pylori* may remain latent for long time and could be associated with gastric cancer (10). Therefore, eradication of these forms of *H. pylori* in inevitable.

In this study, we induced the morphological conversion of *H. pylori* from spiral to coccoid form by *in vitro* exposure to different concentrations of metronidazole, amoxicillin and clarithromycin and determined the type of coccoids as well as their viability and susceptibility to antibiotics. Our observations are important for further studies on the biological behavior and potential pathogenicity of *H. pylori* coccoids and *in vivo* management of their eradication in antibiotic therapies.

## 2. Objectives

The aim of this study was to determine the *in vitro* morphological and bactericidal effects of metronidazole, amoxicillin and clarithromycin on *H. pylori*.

## 3. Materials and Methods

### 3.1. Bacterial Strain and Culture Conditions

In this study, *H. pylori* standard 26695 strain was used. The strain was cultured on Brucella agar (Merck, Germany) containing campylobacter selective supplement (5 mg/L, Merck), trimethoprim (0.25 mg/L), amphotericin B, sheep blood (5%), and 7% fetal calf serum (Sigma, USA). After 72 hours incubation at 37°C in microaerophilic condition (5% O_2_, 85% N_2_, 10% CO_2_) using Mart® microbiology, and the Anoxomat ™ (MART) system (Anoxamat, Lichtenvoorde, Netherlands), the bacterial growth was tested and confirmed as *H. pylori* by Gram staining, urease and oxidase tests. The isolate was stored in brain heart infusion (BHI) broth with 20% glycerol and 1% L-cystein at -70°C until further usage.

### 3.2. Determination of Minimal Inhibitory Concentration (MIC)

MICs of metronidazole, amoxicillin and clarithromycin were determined by E-test strips (AB Biodisk, Solna, Sweden). Briefly, the bacterial suspensions (NO. 3 McFarland standard tube) were prepared and then spread on Muller Hinton agar containing 7% sheep blood and 7% fetal calf serum. One E-test strip was placed on the surface of each cultured plate. After 72 hours, MICs were determined according to manufacturer’s instruction (www.abbiodisk.com).

### 3.3. Induction of Coccoid Forms

Fresh colonies (at 72 hours) of *H. pylori* on Brucella agar (BA) were harvested and confirmed as spiral forms by Gram staining. Bacteria were suspended in Brucella broth (10% fetal calf serum and 2% L-Cystein) (Merck, Germany) and incubated overnight at 37°C in a microaerophilic environment with shaking at 100 rpm. The turbidity of bacterial suspension was adjusted to NO. 3 McFarland, and aliquots were dispensed into ten tubes, each of which contained 5 mL of 109 CFU/mL bacteria.

In three-tube groups, metronidazole, amoxicillin, and clarithromycin, in MIC, 1/2 MIC and 2X MIC were added, respectively. The control tube contained spiral forms without any antibiotics. We examined the samples each 24 hours during 6 days, by Gram staining and observed them by camera light microscopy (Digital DP 72-BX 51, Olympus, Japan) at a magnification of X1000. We screened 100 bacteria per field for their morphology and size of cells. When coccoid forms of *H. pylori* were seen in over 90% of bacteria and their viability was evaluated.

### 3.4. Membrane Integrity and Viability Assay of Cells

Bacterial suspensions in Brucella broth comprised of 90 - 100% coccoid forms of *H. pylori* were centrifuged at 1000 g for 10 minutes and washed twice with PBS (phosphate-buffered salin, pH = 8). The pellets of bacterial suspensions under various concentration and different antibiotics were adjusted to 106 bacteria per milliliter and mixed with 3 μM of 1 mg/mL propidium iodide (PI) fluorescent stain (Sigma, USA). Bacterial cells containing PI stain were incubated for 10 minutes at 4°C.

Analysis was performed with a fluorescence-activated cell sorting (FAC) Scan Calibur flow 140 cytometer (BD, Becton Dickinson, USA). PI stain is a fluorescent dye that penetrates through the uncompleted membrane of dead cells. Forward and side scatter parameters (both in a logarithmic settings) were measured. Red fluorescence of 10000 bacteria was analyzed with FL2 plot. Histogram of the gated bacteria was considered as the percentage of dead and alive cells.

#### 3.4.1. Positive and Negative Controls

Fresh and without antibiotic bacterial suspensions in broth were used as live cells. Bacteria were killed with hypochlorite (5%) and used as dead cells.

### 3.5. Antibiotic Susceptibility Test of Coccoids

Metronidazole, amoxicillin and clarithromycin (Sigma, USA) were added to the broth cultures (contained 100% viable coccoid forms of *H. pylori*) at concentrations of MIC (1, 0.016 and 0.25 µg/mL respectively) and 2X MIC (2, 0.032, and 0.5 µg/mL respectively). After 24 hours, the susceptibility of viable coccoids to each antibiotic was analyzed by flow cytometry assay.

### 3.6. Statistical Analysis

Analysis of data was performed using SPSS version 20 by one way Analysis of variance (ANOVA) and Post Hoc (LSD) tests. A value of P < 0.05 was considered statistically significant.

## 4. Results

### 4.1. Effects of Antibiotics on the Conversion Rate of H. pylori

**Figure 1.**
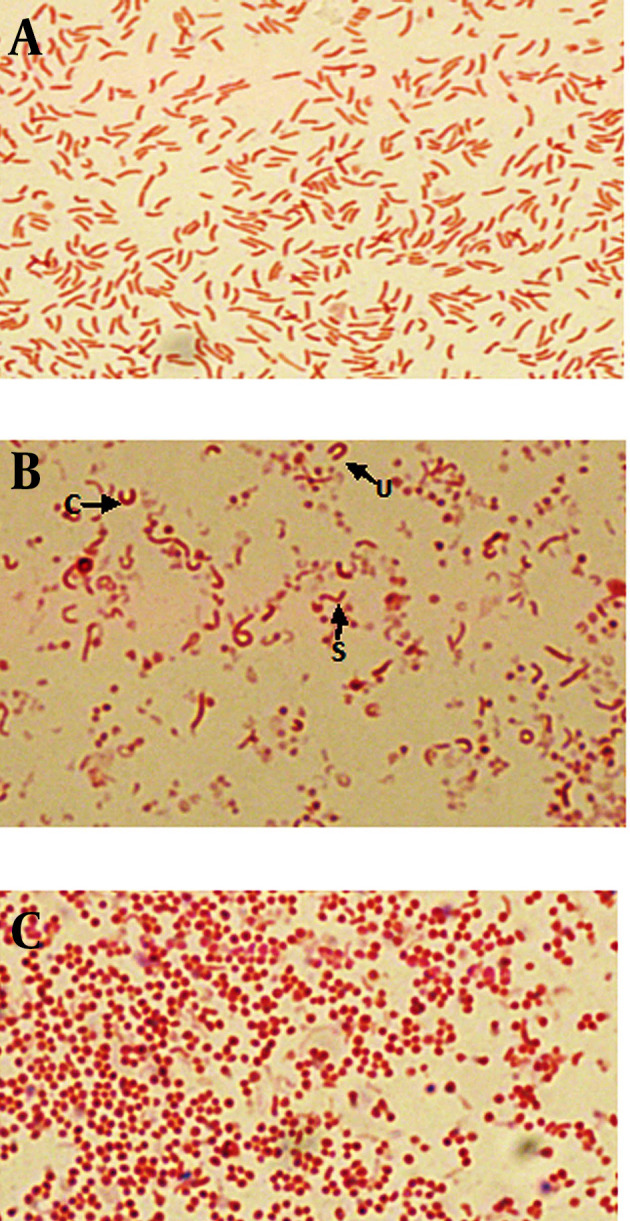
Microscopic Images of Gram-Stained *H. pylori *Under the Light Microscope Showing Different Stages of the Morphologic Conversion (a) pure spiral forms during 24 hours of incubation; (b) mixed forms (S, U, C and coccoid) after exposure to antibiotics; (c) pure induced coccoid forms after 6 days.

Population analyses were performed with the untreated cultures as well as cultures exposed to metronidazole, amoxicillin and clarithromycin, at concentration of 1/2 MIC, MIC and 2X MIC of each antibiotic. The MICs of metronidazole, amoxicillin and clarithromycin were 1, 0.016 and 0.25 μg/mL, respectively. Also the 1/2 MICs were 0.5, 0.008, 0.125 μg/mL and 2X MICs were 2, 0.032 and 0.5 μg/mL, respectively. Gram staining showed that at the initiation of the experiment (24 hours) in untreated broth culture, bacteria were pure spiral (100%) (Figure 1a). During the time (after 72 hours), mixed morphology was seen (spiral, c, u, and coccoid) (Figure 1b). After 6 days of incubation, the populations of cells was pure coccoid forms (100%, Figure 1c). 

**Figure 2.**
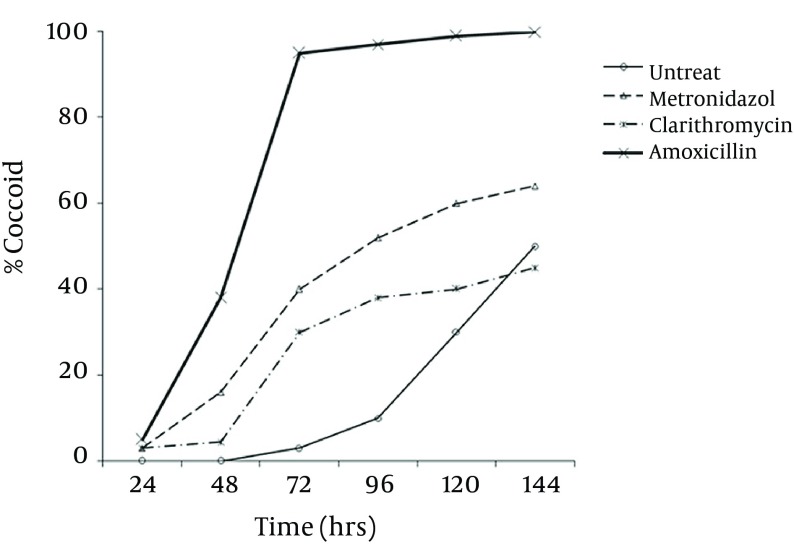
Percentage of Induced Coccoid Forms of *H. pylori *After Exposure to Three Antibiotics at sub (1/2) MIC Concentrations During 24 - 144 Hours

Microscopic study showed two different types of coccoids. One type had 0.5 - 0.8 μm diameter and others were smaller (< 0.3 μm). As shown in Figure 2, it was possible to induce the coccoid forms of *H. pylori *with the applied concentrations of metronidazole, amoxicillin and clarithromycin. A remarkable result was that all three antibiotics generated significant coccoid forms at 1/2 MIC after 72 hours exposure of bacteria (30 - 99.9%). 

The highest rate of induced coccoids was 99.9% after 6 days, related to amoxicillin with 0.008 μg/mL concentration. The rate of induced coccoid forms by metronidazole was 60% at 1/2 MIC after 72 hours. Clarithromycin produced a lower rate of coccoids (40%).

### 4.2. Effects of Antibiotics on the Membrane Integrity and Viability of H. pylori

**Figure 3.**
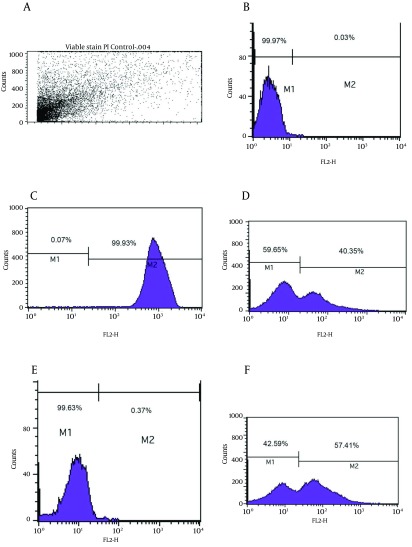
The Flow Cytometry Analysis A) light scatter of gated *H. pylori* cells; B) positive control (Live *H. pylori*); C) dead hypochlorite-treated *H. pylori* as negative control. M1, percentage of alive bacteria; M2, percentage of dead cells. D) Percentage of induced coccoids by metronidazole; E) percentage of induced coccoids by amoxicillin; F) percentage of induced coccoids by clarithromycin.

As shown in Figure 3e, the viability analysis by flow cytometry showed that tubes with amoxicillin at concentration of 0.008 μg/mL (1/2 MIC) had the greatest significant population (99.9%) of viable coccoids compared to two other antibiotics (P = 0.01). The highest rate of induced coccoids occurred after 72 hours and in sub-MICs of antibiotics. Flow cytometry results showed that both types and concentrations of antibiotics affected the rate of viable coccoid induction. Figure 3, sections d and f showed that clarithromycin and metronidazole induced significantly lower rates of viable coccoid forms than amoxicillin (42.5% - 59.65%). 

Microscopic observation showed that the majority of induced coccoids by amoxicillin had small sizes (< 0.3 μm).

### 4.3. Antibiotic Susceptibility of Coccoid Forms

**Figure 4.**
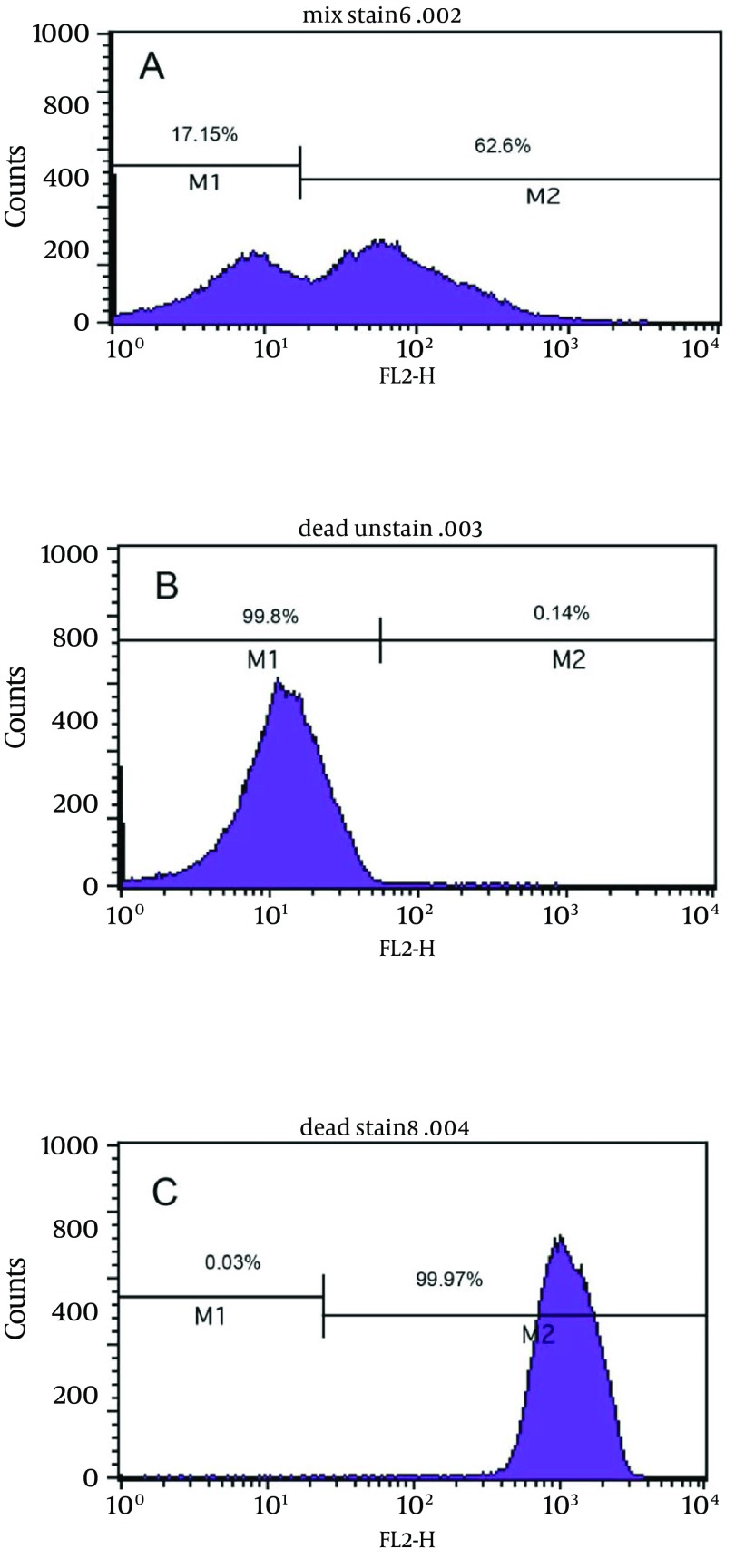
The Flow Cytometry Analysis A) bactericidal effect of metronidazole on viable coccoids; B) bactericidal effect of amoxicillin on viable coccoids; C) effect of clarithromycin on viable coccoids.

Figure 4a shows the bactericidal effect of metronidazole at 2X MIC (2 µg/mL) on viable coccoids by flow cytometry; after 24 hours, 62.6% of cells were killed. Results showed that clarithromycin at MIC and 2X MIC (0.25 and 0.5 µg/mL respectively) had the highest bactericidal effects on viable coccoid forms of *H. pylori *(80 - > 90%) (Figure 4c). Treatment with MIC and 2X MIC of amoxicillin did not inhibit the viable coccoid forms (Figure 4b). 

## 5. Discussion

Increasing reports showed that *H. pylori* can convert from spiral to coccoid form under adverse condition such as *in vitro* exposure to antimicrobial agents (11, 12). During the period of *H. pylori* eradication therapy, some of the organisms may transform into coccoid forms and selectively become resistant to the antibacterial drugs and survive for long time in the gastric environment. These forms possibly contribute to the treatment failures and relapses of infections (13, 14). The investigation of VBNC coccoid forms and their behaviors in stressful environments is important to clarify the pathogenesis of these forms of *H. pylori*. The stress treatments used in this study were antibiotics including metronidazole, amoxicillin and clarithromycin. These antibiotics were chosen because they are typically used for eradication of the *H. pylori* infection.

Our results showed that all three antibiotics could induce the coccoid forms of *H. pylori*. As shown in Figure 1, the highest rate of morphological conversion was related to amoxicillin at concentration of 0.008 μg/mL (1/2 MIC) after 72 hours. The lowest rate of induced coccoids was contributed to clarithromycin. Berry et al. demonstrated that amoxicillin at concentrations close to the MIC could develop the coccoid forms of *H. pylori, *but they did not discriminate the viability of cells (14). We confirmed the viability of induced coccoids after exposure to each antibiotic. Amoxicillin acts on cell wall penicillin binding proteins (PBPs) and may role as an important stress factor for the morphologic changes by expression induction some peptidase enzymes genes that result in modification of the cell wall compositions in coccoid forms (15). 

To confirm the viability and integrity of the induced coccoids, there are various methods (16-18). We used the rapid, simple and reliable flow cytometry method for bacterial viability assay. Sarafnejad et al. (19) also used flow cytometry assay for assessment of *H. pylori* viability. They stained the viable cells by rhodamin123 and concluded that this method was rapid, sensitive and reliable especially for fastidious and nonculturable bacteria. Their stain was different from ours. We used PI stain that was easier to use and more cost effective than Rh123. Berry et al. determined the survival and susceptibility of coccoid forms of *H. pylori* by colony count method. Their study reported the viable nonculturable coccoids as dead cells (14). Many authors (9, 11) have demonstrated that different concentrations of amoxicillin, erythromycin, metronidazole, clarithromycin and bismuth subsalicylate were able to induce the coccoid forms of *H. pylori* at different times of exposure. Their different results might be due to the strains specificity and various methods of evaluation.

In this study, we tested the susceptibility of viable coccoids to the three antibiotics and showed that clarithromycin had the highest bactericidal effect on these types of cells; hence, the MIC of this antibiotic for spiral and coccoid forms was 0.25 µg/mL (Figure 4c). Similar results were derived from metronidazole. It seems that protein synthesis inhibitor antibiotics such as clarithromycin and DNA inhibitor antibiotics such as metronidazole had appropriate bactericidal effects on coccoid forms of *H. pylori*. Amoxicillin had the greatest *in vitro* bactericidal effect on bacillary forms of *H. pylori; *however, in 2X MIC it had no bactericidal effect on coccoids (Figure 4b). In agreement with our results, Berry et al. showed that coccoid forms of *H. pylori *were resistance to 10X MIC of amoxicillin (14). 

In addition to the potent *in vitro* effect of amoxicillin against *H. pylori*, this antibiotic has a little *in vivo* bactericidal effect (14). Transition to coccoid forms correlates with accumulation of the N-acetyl-D-glucosaminyl-β(1,4)-N-acetylmuramyl-L-Ala-D-Glu (GM-dipeptide) motif. This modification may have a role in resistance to bactericidal agents such as amoxicillin that act on the cell wall such as amoxicillin (20). Costa et al. (20) demonstrated that the activation of glutamyl-diaminopimelate endopeptidase during the morphological transition in of *H. pylori* was similar to sporulation in *Bacillus sphaericus*. This phenomenon results in the genesis of resistant coccoid forms and endospores in *H. pylori* and *B. sphaericus* respectively. High resistance of these forms may be contributed to different compositions of the cell wall in coccoids and loss of antibiotic targets in these forms. Moreover, the dormant states of coccoid forms that are metabolically quiescent were resistant to antibiotics that act on replicative cells.

In conclusion, our study revealed that different antibiotic concentrations close to the MIC exposed to *H. pylori,* can induce the morphologic transformation from spiral to coccoid forms. The greatest induction effect was seen by amoxicillin. Viable coccoids were resistant to amoxicillin. Therefore, for successful therapy, it may be important not only to eradicate the spiral and coccoid forms of *H. pylori*, but to prevent the induction of coccoid forms. We suggest that susceptibility testing alone may not be sufficient to determine the clinical effects of antibiotics on *H. pylori* infection. A further attention is necessary for administration of anti *H. pylori* treatment drugs in chronic gastritis infections.
